# COVID-19: eine Chance zur Digitalisierung der Lehre? Erfahrungsbericht und Ergebnisse einer Umfrage zur digitalen Lehre im Bereich Anästhesiologie, Intensiv-, Notfall-, Schmerz- und Palliativmedizin an der Universität Leipzig

**DOI:** 10.1007/s00101-021-01016-4

**Published:** 2021-08-02

**Authors:** Gunther Hempel, Andreas Weissenbacher, Sebastian N. Stehr

**Affiliations:** Klinik und Poliklinik für Anästhesiologie und Intensivtherapie, Universitätsklinikum Leipzig AöR, Liebigstraße 20, 04103 Leipzig, Deutschland

**Keywords:** Medizinstudium, Curriculum, Online, Podcast, Prüfung, Medical studies, Curriculum, Online, Podcast, Assessment

## Abstract

**Hintergrund:**

Die SARS-CoV-2-Pandemie hat die Universitäten vor große Herausforderungen gestellt. Innerhalb kürzester Zeit galt es, Lehrveranstaltungen zu digitalisieren. Dies betraf auch den Bereich Anästhesiologie, Intensiv‑, Notfall‑, Schmerz- und Palliativmedizin an der Universität Leipzig.

**Fragestellung:**

Ziel der fragebogengestützten Untersuchung war es herauszufinden, welche Veranstaltungen aus Sicht der Studierenden am ehesten digitalisiert werden können, und welche technische Infrastruktur die Lehrenden bei der Digitalisierung jeweils bestmöglich unterstützt.

**Material und Methoden:**

Für die digitale Durchführung der Lehrveranstaltungen wurden Videopodcasts, digitale Lernmaterialien, Lehrfilme und Videokonferenzen genutzt. Je nach Veranstaltung wurden verschiedene dieser Angebote kombiniert. Darüber hinaus wurde ein Diskussionsforum für den Austausch zwischen Lehrenden und Studierenden etabliert. Zur Bewertung der Inhalte erfolgte im Anschluss eine Onlineevaluation.

**Ergebnisse:**

An der Befragung haben 82 Studierende teilgenommen. Als effektivste Angebote zur Wissensvermittlung wurden die Videopodcasts der Vorlesung (45,1 %) sowie der elektronische Unterricht am Krankenbett (34,1 %) bewertet. Insbesondere die Vorlesungen könnten nach Meinung von 92,7 % der befragten Studierenden auch dauerhaft digital ersetzt werden. Knapp 90 % haben die digitalen Lehrangebote der Klinik mit einer Gesamtnote von 1 oder 2 bewertet.

**Diskussion und Zusammenfassung:**

Im Zuge des digitalen Semesters ließen sich einzelne Lehrformate unterschiedlich gut digitalisieren: Vorlesungen können aus Sicht der Studierenden auch langfristig besonders gut digital abgebildet werden, währenddessen die Digitalisierung des Unterrichts am Krankenbett bisher nicht adäquat möglich ist.

**Zusatzmaterial online:**

Die Online-Version dieses Beitrags (10.1007/s00101-021-01016-4) enthält weitere Abbildungen.

## Abstract (en)

**Background:**

The SARS-CoV‑2 pandemic has been a major challenge for graduate education. Teaching had to be digitalized within a very short time. This also affected the areas of anesthesiology, intensive care, emergency, pain and palliative care at the Department of Anesthesiology and Intensive Care Medicine at the University of Leipzig.

**Objective:**

The aim of this questionnaire-based survey was to find out which courses can be digitalized from the students’ point of view and which forms of teaching are associated with obstacles. In addition, we examined which technical infrastructure supports digitalization best.

**Material and methods:**

In the course of digitalization the lecture series in the areas of palliative care and pain medicine had to be revised but also digital alternatives for seminars, simulation courses and bedside teaching had to be created. Video podcasts, digital learning material, educational films and video conferences were used for the digital implementation of the courses. Depending on the course, different digital methods were combined. In addition, a discussion forum for the exchange between faculty and students was established. An online evaluation was then carried out to assess the content.

**Results:**

A total of 82 4th and 5th year medical students took part in the survey. More than 60% of students rated the learning effect of digital courses as “high” or “very high”. Video podcasts of the lectures (45.1%) and digital bedside teaching (34.1%) were rated as the most effective ways of imparting knowledge. In particular, 92.7% of the surveyed students believed that the lectures could be replaced digitally on a permanent basis. For bedside teaching (3.7%) and emergency simulation course (1.2%) this is far less the case. In the majority of cases (56.1%), students needed 30–90 min daily for the preparation and post-processing of the contents. Just under 90% gave the digital courses offered by the hospital an overall grade of 1 or 2 (on a scale from 1 = best to 6 = worst).

**Conclusion:**

The SARS-CoV‑2 pandemic posed major challenges for graduate teaching. At the same time, however, it also helped to overcome often long-standing hurdles to the digitalization of teaching. In the course of the digital semester, different teaching formats could be digitalized to varying degrees: Lectures can be digitally reproduced particularly well from the students’ perspective, whereas the digitalization of bedside teaching has not been possible in most cases.

## Einleitung

Das Leben in Deutschland wurde durch die SARS-CoV-2-Pandemie seit dem Frühjahr 2020 sprichwörtlich auf den Kopf gestellt. Lehrveranstaltungen an Universitäten waren dabei von tiefgreifenden Änderungen betroffen, galt es doch, die oft seit vielen Jahren etablierten Veranstaltungen in kurzer Zeit so anzupassen, dass eine Onlinedurchführung möglich war. Aus den hierbei gemachten Erfahrungen und den Ergebnissen einer Befragung betroffener Studierender sollen in der vorliegenden Arbeit Möglichkeiten der digitalen Lehre im Bereich Anästhesiologie, Intensiv-, Notfall-, Schmerz- und Palliativmedizin (AINSP) diskutiert werden.

## Hintergrund

Bei der Meldung erster Fälle von Pneumonien unbekannter Ursache in der chinesischen Region Wuhan Ende 2019 [30] hätte wohl kaum einer für möglich gehalten, dass sich daraus wenige Wochen später eine Pandemie entwickeln würde, die auch die Gesundheitssysteme und das öffentliche Leben in Europa vor ungeahnte Herausforderungen stellt. Im Zuge der dadurch notwendigen Kontaktbeschränkungen und Hygienemaßnahmen wurde durch die Kultusministerkonferenz der Länder im Hinblick auf die Universitäten empfohlen, die Sommersemester regulär starten zu lassen, jedoch die Lehrveranstaltungen primär digital durchzuführen [15]. Diesem Beschluss folgte in Abstimmung mit der Landesrektorenkonferenz auch die Medizinische Fakultät der Universität Leipzig [23]. Vordergründiges Ziel war es daher, die bereits lange etablierten Präsenzlehrveranstaltungen zeitnah so umzubauen, dass eine digitale Durchführung nicht nur möglich, sondern auch gewinnbringend für alle Beteiligten ist. Für die Klinik und Poliklinik für Anästhesiologie und Intensivtherapie der Universität Leipzig bedeutete dies für das Sommersemester 2020, die Vorlesungsreihen in den Querschnittsbereichen (QSB) 13 und 14 zu digitalisieren, aber auch digitale Alternativen für Seminare (QSB 13), Simulationskurse (QSB 8) und den Unterricht am Krankenbett („bedside teaching“) zu schaffen. Die Auswirkungen dieser Anpassungen der Lehrveranstaltungen und mögliche Konsequenzen für zukünftige Semester sollten im Anschluss mithilfe einer Onlinebefragung unter den betroffenen Studierenden evaluiert werden. Konkret galt es dabei zu klären, welche Lehrformate sich aus studentischer Sicht am ehesten gut und effektiv digital abbilden lassen, wo digitale Inhalte ggf. auch über die Pandemie hinaus sinnvoll eingesetzt werden können, und wo die rein digitale Lehre zur Wissensvermittlung an ihre Grenzen stößt.

## Methodik und Durchführung

### Technische Infrastruktur

**Figure 1:**
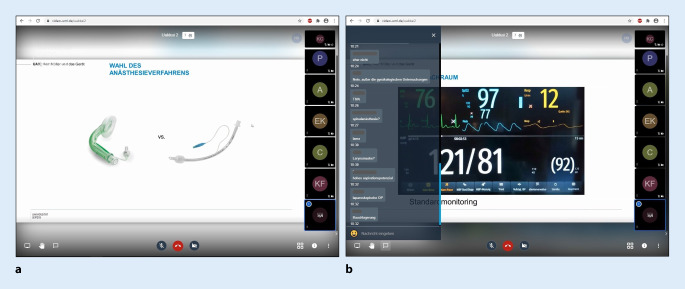

Bis zum Beginn der Pandemie hatten alle Studierenden über ein zentrales Studierendenportal zugangsgeschützt Zugriff auf eine Vielzahl von *pdf-Dateien von Vorlesungsfolien sowie einzelne Videopodcasts und Lehrvideos. Beim Studierendenportal der Medizinischen Fakultät (https://student.uniklinikum-leipzig.de/) handelt es sich um eine Eigenentwicklung, in der die Studierenden nach einem individuellen Log-in neben digitalen Lehrmaterialien auch alle weiteren für das Studium relevanten Informationen finden (Klausurergebnisse, Stundenpläne, Evaluationen usw.). Die Bereitstellung und Verknüpfung der digitalen Lehrmaterialien erfolgten auch weiterhin über dieses Portal als zentrale Lernplattform (Zusatzmaterial online: Abb. 1). Jedoch wurden seitens der Medizinischen Fakultät Leipzig kurzfristig weitere Ressourcen zur Unterstützung der digitalen Lehre zur Verfügung gestellt. Neben dem reinen Ausbau der verfügbaren Serverkapazität wurde u. a. basierend auf der Open-Source-Software phpBB® (phpBB Limited; GNU GPL v2.0) ein Forum etabliert (https://www.forum-uml.de/). In diesem war und ist es möglich, Themen zwischen Studierenden und Lehrenden zu besprechen, wobei jedes Fachgebiet (bzw. jeder Leistungsnachweis nach ÄApprO) ein eigenes Unterforum erhielt. Alle Studierenden bekamen automatisch einen Account, der mit den bestehenden individuellen Log-in-Daten für das Studierendenportal verknüpft war. Des Weiteren wurde allen Lehrbeauftragten automatisch und weiteren interessierten Lehrenden auf Antrag ein Account erstellt (Zusatzmaterial online: Abb. 3 und 4). Ziel war es hier, ein asynchrones Interagieren und Zusammenarbeiten von Studierenden und Lehrenden über die einzelnen Lehrveranstaltungen hinweg zu ermöglichen [20, 25]. Neben dem Forum wurde auf einem gesonderten Server eine Open-Source-Videokonferenzsoftware auf der Basis von Jitsi™ (8 × 8, Inc,; Campbell, CA, USA) bereitgestellt (https://www.video-uml.de/), mit der es für alle Lehrenden möglich war, ohne technische Vorkenntnisse Videokonferenzen im Rahmen von Lehrveranstaltungen durchzuführen und diese zur effektiven Wissensvermittlung im direkten Austausch mit den Studierenden in kleineren und größeren Gruppen einzusetzen [13]. Im weiteren Verlauf erhielten alle Lehrenden darüber hinaus die Möglichkeit, die Webkonferenzsoftware BigBlueButton (BigBlueButton Inc.; Ontario, Kanada) für Lehrveranstaltungen zu nutzen (https://www.webkonferenz-uml.de/).

### Vorlesungsreihen Palliativmedizin (QSB 13) und Schmerzmedizin (QSB 14)

Ausgehend von diesen technischen Möglichkeiten wurden das Vorgehen und die verschiedenen Optionen für die bestmögliche digitale Durchführung der einzelnen Lehrveranstaltungen entwickelt. Am einfachsten erschien hierbei die alternative Durchführung der Vorlesungsreihen (QSB 13 und QSB 14). Diese wurden den Studierenden neben den *pdf-Dateien der Vortragsfolien im Einklang mit bisherigen Studienergebnissen jeweils als Videopodcasts/Screencasts zur Verfügung gestellt [21]. Die Erstellung der Screencasts erfolgte mithilfe der Software Camtasia Studio (TechSmith, Okemos, MI, USA), wobei die Videodateien anschließend zugangsgeschützt im Studierendenportal zur Verfügung gestellt wurden.

### QSB 13 – Palliativmedizin

Das Seminar des QSB 13 Palliativmedizin, welches sonst an 5 aufeinanderfolgenden Tagen á 45 min für jeweils eine Kursgruppe von rund 15 Studierenden individuell stattfindet, wurde durch ein komplett digitales Flipped-Classroom-Konzept ersetzt. Hierbei handelt es sich um eine Lehrmethode, bei der die üblichen Aktivitäten innerhalb und außerhalb des Hörsaals „umgedreht“ werden [19, 27]. Die Studierenden eignen sich die verschiedenen digital zur Verfügung gestellten Lehrinhalte selbstorganisiert an. Die eigentliche Präsenzveranstaltung wird dann zur gemeinsamen Vertiefung des Gelernten genutzt. An den ersten 4 Tagen haben die Studierenden daher unter Berücksichtigung zuvor online veröffentlichter Lernziele selbstständig wichtige palliativmedizinische Themen erarbeitet. Hierzu wurden im Studierendenportal verschiedene Videos von Patientengesprächen und klinischen Untersuchungen von 5–15 min Dauer zur Verfügung gestellt. Tagesspezifisch gab es ergänzende Arbeitsblätter und Aufgabenstellungen, die als roter Faden dienen sollten. Am letzten Kurstag erfolgte dann für jede Kursgruppe eine gemeinsame Videokonferenz von 45 min Dauer mit den Lehrkräften der Palliativmedizin. Hier wurden die über die Woche verteilten Aufgaben ausgewertet, nochmals Patientenfälle vorgestellt und letzte offene Fragen geklärt.

### QSB 8 – Notfallmedizin

Eine größere Herausforderung stellt die digitale Durchführung des Notfallsimulationskurses als Teil des Querschnittsbereiches 8 „Notfallmedizin“ dar. Hierbei handelt es sich um einen 3‑tägigen Kurs, der eigentlich für jeweils 90 min komplett im Simulationszentrum der Klinik durchgeführt wird. Ziel ist es, neben der praktischen Anwendung der aktuellen Reanimationsleitlinien in Kleingruppen auch Aspekte des Crew Resource Management (CRM) zu erleben und in einem Debriefing vermittelt zu bekommen. Dieser Kurs wurde ebenfalls im Sinne eines Flipped-Classroom-Konzeptes umgewandelt. Die rund 15 Studierenden der jeweiligen Kursgruppe bekamen hierzu 4 Screencasts zu den Themen Basic Life Support, Advanced Life Support (Teil 1 + 2) sowie CRM im Studierendenportal zur Verfügung gestellt. In diesen jeweils 30- bis 45-minütigen Beiträgen wurden die Inhalte, abgestimmt auf die Kurslernziele, von verschiedenen Referentinnen und Referenten der Klinik aufbereitet und zusammengefasst (Zusatzmaterial online: Abb. 5). Für besonders interessierte Studierende gab es darüber hinaus ergänzende Videobeiträge, interessante Publikationen und eine Verlinkung zu den aktuellen Leitlinien des European Resuscitation Council [6]. Ähnlich wie bei den Seminaren der Palliativmedizin gab es am letzten Kurstag eine gemeinsame 90-minütige Videokonferenz zwischen allen Studierenden der Kursgruppe und einer Dozentin bzw. einem Dozenten. Hierbei wurden zur Wissensvertiefung noch einmal verschiedene Fälle virtuell durchgespielt und mögliche offene Fragen abschließend geklärt.

### Unterricht am Krankenbett

Als letzte große Herausforderung galt es, den Unterricht am Krankenbett (Bedside teaching) im Bereich der Anästhesiologie und Intensivmedizin bestmöglich zu digitalisieren. Der Kurs findet regulär an 10 Terminen á 90 min direkt im OP oder auf der Intensivstation statt. Hierbei wird die Kursgruppe von 15 Studierenden in verschiedene Untergruppen á 4 bis 5 Studierende aufgeteilt, die am Ende meist in 1:1-Betreuung im OP unterrichtet werden. Die digitale Umsetzung erfolgte auf verschiedenen Wegen. Noch vor Beginn des Semesters wurden entsprechend dem Lernzielkatalog für jeden einzelnen Kurstag Patientenfälle erarbeitet und in Präsentationsform im Einklang mit den jeweiligen Lernzielen aufbereitet. Zusätzlich wurden für verschiedene grundlegende praktische Fertigkeiten, die auch außerhalb der Anästhesiologie Bedeutung haben, kurze Lehrvideos erstellt und durch erläuternde Skripte ergänzt (beispielhaft im Zusatzmaterial online: Abb. 1 und 2). Die eigentlichen Kurse fanden dann zu den regulären Praktikumszeiten in Form einer Videokonferenz für jeweils eine Kursgruppe statt. Entsprechend dem veröffentlichten Praktikumsplan wurde an jedem Tag ein spezifisches Thema (z. B. Prämedikation, Atemwegsmanagement, Allgemeinanästhesie) anhand eines Fallbeispiels durch eine Dozentin bzw. einen Dozenten vorgestellt. Ziel war es dabei, in jedem Termin die Studierenden in der Videokonferenz aktiv in die Diskussion und Erarbeitung des Themas einzubeziehen. Dies erfolgte sowohl per Audiokommentar, aber auch über die in die Videokonferenzplattform integrierte Chat-Funktion. Abb. 1 zeigte dabei beispielhaft einmal 2 Screenshots aus einer laufenden Videokonferenz.

**Figure 2:**
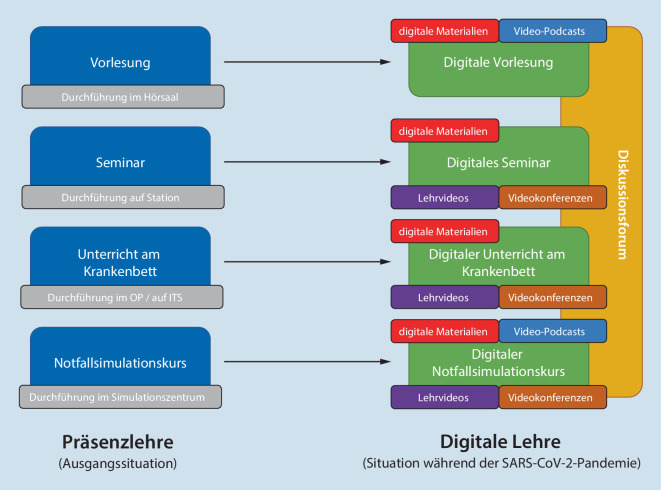

Eine Übersicht über die grundlegenden Anpassungen in den einzelnen Lehrveranstaltungen liefert Abb. 2.

### Evaluation

Nach Abschluss des digitalen Sommersemesters wurden alle Studierenden des 4. und 5. Studienjahres zur freiwilligen Teilnahme an einer Onlineevaluation der Lehrveranstaltungen der Klinik und Poliklinik für Anästhesiologie und Intensivtherapie eingeladen. Die Befragung bestand aus insgesamt 11 Single‑/Multiple-Choice- und 2 Freitextfragen. Der Fragebogen wurde vorab durch alle an der studentischen Lehre beteiligten Kolleginnen und Kollegen der Klinik gemeinsam erstellt, mit der Klinikleitung abgestimmt und durch den Datenschutzbeauftragten der medizinischen Fakultät freigegeben. Aufgrund der reinen Erhebung von Evaluationsdaten bestand nach Rücksprache mit der hiesigen Ethikkommission zudem keine darüber hinausgehende Beratungspflicht. Nach einem Pretest unter Ärztinnen und Ärzten in Weiterbildung der Klinik wurde die eigentliche Befragung Anfang Juli 2020 online mithilfe der Evaluationssoftware EvaSys v8.0© (Fa. Electric Paper Evaluationssysteme GmbH, Lüneburg, Deutschland) durchgeführt. Die Studierenden erhielten dazu einen individuellen Link per E‑Mail zugesandt und wurden ebenfalls per E‑Mail einmalig an die Teilnahme an der Evaluation erinnert. Die Auswertung und Aufbereitung der erhobenen Daten erfolgten mithilfe des durch EvaSys v8.0© automatisch erstellten *pdf-Reports sowie der Software GraphPad Prism v8.4.3 für Windows (Fa. GraphPad Software Inc., San Diego, CA, USA).

## Ergebnisse

Im Sommersemester 2020 wurden für den Studiengang Humanmedizin insgesamt etwa 1400 Videos erstellt und hochgeladen (im Jahr davor waren es etwa 70). Die Videos (Lehrvideos, Podcasts etc.) wurden ca. 330.000-mal abgerufen und erzeugten damit einen Datentransfer von mehr als 50 Terabyte (im Vorjahr waren es rund 17.500 Zugriffe). Im eigens erstellen Diskussionsforum wurden im Verlauf des Semesters etwa 3200 Beiträge in 600 Themen veröffentlicht. Die durchgeführten Videokonferenzen hatten eine kumulierte Dauer von etwa 150 Tagen (3600 h).

**Figure 3:**
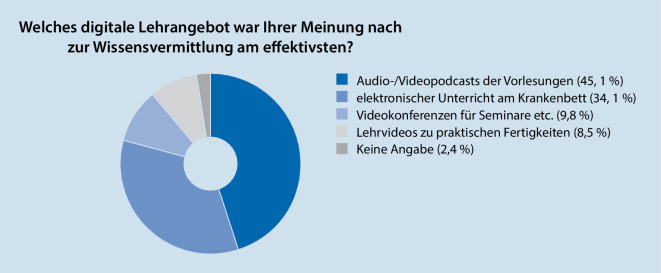

An der freiwilligen Befragung am Ende des Semesters haben insgesamt 82 Studierende von möglichen 596 Studierenden der beiden Studienjahre teilgenommen (Rücklaufquote 13,8 %). 41,5 % der Befragten gehörten dem 4. Studienjahr an, 56,1 % dem 5. Studienjahr – 2,4 % der Studierenden machten hierzu keine Angaben. Als effektivste Lehrangebote zur Wissensvermittlung wurden von den Befragten die Audio‑/Videopodcasts der Vorlesung (45,1 %) sowie der elektronische Unterricht am Krankenbett (34,1 %) bewertet (Abb. 3).

**Figure 4:**
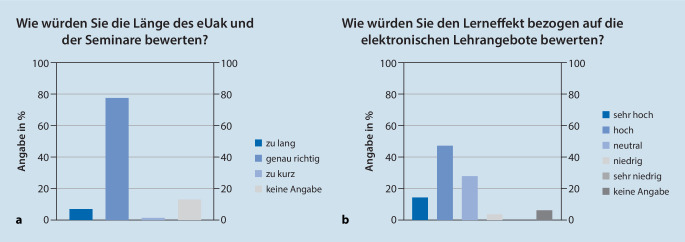

Die Dauer des elektronischen Unterrichts am Krankenbett und der digitalen Seminare wurde von rund 80 % der Studierenden als genau richtig eingeschätzt (Abb. 4a). Den Lerneffekt der elektronischen Lehrangebote bewerteten gut 60 % der Studierenden mit „hoch“ bzw. „sehr hoch“ (Abb. 4b).

**Figure 5:**
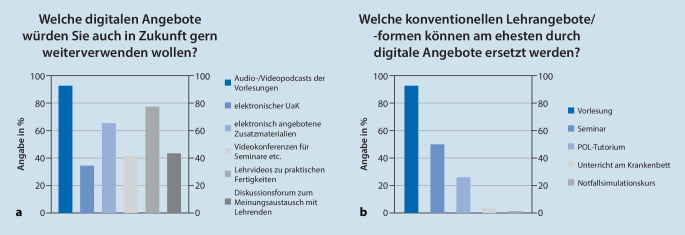

Bezüglich der Frage, welche digitalen Lehrangebote die Studierenden auch in Zukunft gern weiterverwenden wollen, sprechen sich diese v. a. für Audio‑/Videopodcasts der Vorlesungen (92,7 %) sowie für Lehrvideos zu praktischen Fertigkeiten (78,0 %) und die elektronische Bereitstellung ergänzender Zusatzmaterialien (65,9 %) aus (Abb. 5a). Ebenfalls 92,7 % der Studierenden sehen die Vorlesung als eine Lehrform an, die dauerhaft durch digitale Alternativen ersetzt werden könnte. Für den Unterricht am Krankenbett (3,7 %) und den Notfallsimulationskurs (1,2 %) ist das weit weniger der Fall (Abb. 5b).

**Figure 6:**
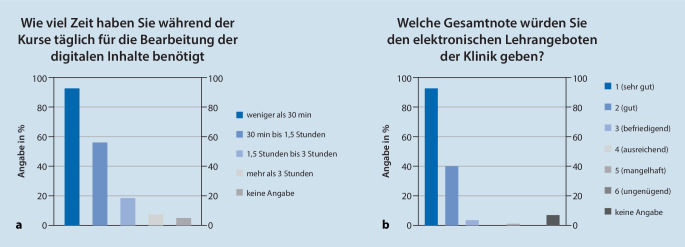

Die Zeit, die die Studierenden täglich für die Vor- und Nachbearbeitung der Inhalte benötigt haben, lag in der Mehrzahl der Fälle (56,1 %) bei 30–90 min (Abb. 6a). Befragt nach einer Gesamtnote für die digitalen Lehrangebote der Klinik, bewerteten knapp 90 % der Studierenden diese mit einer Note 1 oder 2 (Abb. 6b).

## Diskussion

Betrachtet man zuerst einmal die Ergebnisse im Hinblick auf die Fragestellung, so können aus Sicht der Studierenden v. a. Lehrveranstaltungen ohne direkten Patientenkontakt wie Vorlesungen gut digitalisiert werden. Dies gilt sogar über die Pandemie hinaus. Demgegenüber wurde der digitale Unterricht am Krankenbett zwar als effektiv in der Wissensvermittlung angesehen – er ist aus Sicht der Befragten jedoch kein Angebot, was dauerhaft digital durchgeführt werden sollte.

Im Detail zeigt sich also schon fast erwartbar, dass sich einzelne Lehrveranstaltungen in den Augen der Studierenden besser digitalisieren lassen als andere. Gerade unter Berücksichtigung der im Zuge der Pandemie begrenzten Ressourcen – aber auch für die Zeit danach – erscheint es daher besonders wichtig, vorab eine Auswahl zu treffen, um durch die Digitalisierung auch einen wirklichen Mehrwert zu erreichen [28]. Beispielhaft sei hier die Vorlesung genannt, die nach Meinung von über 90 % der Befragten auch künftig gut durch digitale Lehrangebote ersetzt werden könnte. Konzeptionell wichtig erscheint es in diesem Zusammenhang, eine Möglichkeit zu schaffen, die direkte Interaktion mit den Studierenden, die z. B. durch Nachfragen im Hörsaal bei Präsenzveranstaltungen möglich ist, auch digital abzubilden [12]. Bei einer asynchronen Bereitstellung der Vorlesungsaufzeichnungen in Form von Videopodcasts, ist dies z. B. über ein Forum möglich. Im Vergleich zur Kommunikation per E‑Mail wäre hier der Vorteil, dass alle Studierenden die Möglichkeit haben, an der Diskussion teilzunehmen. Bei Durchführung der Vorlesung als Livestream ist je nach Plattform auch eine direkte Interaktion mit den Studierenden über einen Chat oder eine Rückkopplung über in die Vorlesung integrierte Umfragen möglich. Eine entsprechende IT-Infrastruktur vorausgesetzt, scheint uns letztere Variante die perspektivisch bessere Möglichkeit darzustellen, welche wir seit dem Wintersemester 2020/2021 mithilfe der Webkonferenzsoftware BigBlueButton (Fa. BigBlueButton Inc., Ontario, Kanada) auch selbst umsetzen. Die Aufzeichnungen dieser Vorlesungen werden den Studierenden im Anschluss ebenfalls zur Nachbereitung zur Verfügung gestellt. Auffallend ist unserer eigenen Erfahrung nach, dass die Studierenden über den Chat und Wortmeldungen per Audiosignal deutlich mehr mit den Lehrenden interagieren, als dies zuvor in den Präsenzvorlesungen der Fall war. Eventuell ist hierbei im digitalen Umfeld die Hemmschwelle aus Sicht der Studierenden niedriger.

Abgesehen von den Vorlesungen können andere Lehrveranstaltungen wie der Unterricht am Krankenbett oder Simulationskurse, deren Inhalte auf dem Training praktischer Fertigkeiten und der direkten Interaktion im Team beruhen (z. B. Notfallsimulationskurs) aus Sicht von über 90 % der Befragten nicht durch digitale Angebote ersetzt werden. Dies wird auch darin deutlich, dass viele medizinische Fakultäten bestrebt sind, v. a. diese Kurskonzepte möglichst bald wieder im herkömmlichen Format durchführen zu können. Aktuell kann man nur versuchen, aus den vorhandenen Mitteln mit Lehrvideos praktischer Tätigkeiten oder Training an Simulatoren das Maximale herauszuholen [8]. Klar ist aber auch, dass die Konzeption und Produktion didaktisch gut aufbereiteter Lehrvideos ressourcenintensiv sind [14]. Virtuelle Patientinnen und Patienten oder auch komplexe Anwendungen im Bereich der virtuellen Realität (VR) bzw. „augmented reality“ (AR) könnten hier künftig Alternativen darstellen. VR- und AR-Anwendungen sind jedoch aktuell noch nicht flächendeckend, sondern meist nur im Rahmen kleinerer Modellprojekte oder Wahlfächer, im Einsatz [2, 11]. Gerade im Hinblick auf die vielfach geforderte kompetenzorientierte Ausbildung können diese Angebote die Ausbildung an Patienten/Patientinnen unserer Ansicht nach auch langfristig bestenfalls unterstützen, aber keinesfalls ersetzen.

Obgleich Digitalisierung und digital unterstütze Lehre schon seit vielen Jahren in aller Munde sind, stellt sich die Frage, wieso es in der Umsetzung an vielen medizinischen Fakultäten gerade jetzt einen deutlichen Schub gibt? In einer 2018 veröffentlichten Befragung unter Lehrbeauftragten konnte gezeigt werden, dass fehlende personelle und zeitliche Ressourcen als größte Hinderungsgründe für den Einsatz neuer digitaler Lehrmedien/-materialien gesehen werden [29]. Diese Hürden konnten nun offenbar nicht nur in Leipzig durch den SARS-CoV-2-bedingten Zwang zur Umstellung auf digitale Inhalte übersprungen werden. Klar ist, dass die Umwandlung und Digitalisierung etablierter Lehrkonzepte ressourcenintensiv sind und somit auch eine hohe Motivation bzw. ein Wille zu Veränderung gegeben sein muss. Beispielhaft hat z. B. Prof. Jürgen Handke hierzu bereits vor einigen Jahren ein mehrstufiges Konzept veröffentlicht und im eigenen Studiengang umgesetzt [9]. Bisher gab es in Deutschland jedoch noch keine flächendeckenden Konzepte für digitale Lehr- und Lernformate. Vieles basierte auf der Initiative von einzelnen Lehrenden oder auf der Nutzung bestimmter Tools durch Studierende, wobei hier v. a. das Bestehen der Prüfungen als Hauptmotivation anzusehen war („assessment drives learning“) [16]. Um die komplexen Anforderungen möglichst flächendeckend bewältigen zu können, wurde schon vor einigen Jahren eine nationale Initiative „Medizinausbildung im digitalen Zeitalter“ gefordert [7]. Fakt ist, dass die aktuelle SARS-CoV-2-Pandemie das Medizinstudium weltweit beeinflusst hat [22, 24]. Die Frage ist, ob die aktuellen Veränderungen der Startschuss hin zu einer tiefgreifenden Digitalisierung des Medizinstudiums sind, oder ob nach Abklingen der Pandemie alles wieder auf herkömmliche Lehrangebote umgestellt wird [1].

Eine aktuelle Metaanalyse konnte zeigen, dass digital unterstütztes Lernen neben der studentischen Lehre auch in der anästhesiologischen Weiterbildung sinnvoll eingesetzt werden kann [26]. Selbst in Bereichen, die auf den ersten Blick dafür nicht infrage kommen – wie die Vermittlung von Kompetenzen im Bereich der Kommunikation – scheint die Digitalisierung in Form von Blended-Learning-Konzepten Vorteile zu bringen [17]. Es ist also mittlerweile nicht mehr die Frage, ob Digitalisierung der Lehre Vorteile bringen kann, sondern eher wo und wie der Einsatz erfolgen sollte, um den größten Mehrwert zu generieren. Denn unsere Befragung zeigt auf der anderen Seite auch, dass zwangsweise Digitalisierung aller Lehrveranstaltungen kein Allheilmittel ist.

Betrachtet man die Rückmeldungen der beteiligten Lehrenden unserer Klinik, so sind diese nach anfänglicher Skepsis ob des organisatorischen Mehraufwands und der fehlenden direkten studentischen Rückkopplung im Vergleich zur Präsenzlehre mittlerweile vollends von den Vorteilen gezielt eingesetzter digitaler Lehrinhalte überzeugt. Unser eigenes Fazit, welches sich sicherlich auch auf andere Standorte übertragen lässt, ist es daher, in den nächsten Monaten mehr und mehr an hybriden Lehrkonzepten zu arbeiten, die digitale und Präsenzlehre miteinander verknüpfen. So ist geplant, die künftigen Präsenzvorlesungen parallel live zu streamen und nachträglich als Aufzeichnungen den Studierenden zur Verfügung zu stellen. Wir werden versuchen, die Praktika und Seminare unserer Klinik flächendeckend auf ein Flipped-Classroom-Konzept umzustellen [18]. Hierzu werden wir die vorhandenen digitalen Lehrangebote an Podcasts und Lehrvideos weiterentwickeln und kombiniert mit Lerntexten und Aufgaben für die Kursvorbereitung einsetzen. Die eigentlichen Seminare und Praktika sollen dann im Einklang mit den Ergebnissen unserer Befragung in Präsenz stattfinden und einzig und allein der praktischen Anwendung des zuvor erlernten Wissens dienen.

Schaut man in die nahe Zukunft, so sind die direkten Auswirkungen der SARS-CoV-2-Pandemie auf das Medizinstudium weltweit immer noch schwer abschätzbar. Eine Befragung unter Medizinstudierenden in den USA zeigt, dass gut 20 % der Befragten glauben, dass die Pandemie die Auswahl der späteren medizinischen Fachdisziplin beeinflusst [3]. Dies liegt u. a. daran, dass die Studierenden einige Fachgebiete pandemiebedingt gar nicht richtig kennenlernen können. Inwieweit sich dies auf den Ärztinnen- und Ärztemangel in bestimmten Fachgebieten in Deutschland auswirken wird, bleibt abzuwarten. Gerade die Studierenden der höheren Fachsemester fühlen sich entsprechend einer Befragung in Großbritannien bezüglich des fließenden Übergangs in den Arztberuf schlechter vorbereitet [4] oder gar durch angepasste Prüfungen im Zuge der Pandemie zu schnell in den Beruf gedrängt [10]. Bei der Wiederaufnahme des Studienbetriebs sollten die Meinungen der Studierenden daher bereits bei der Konzeption eines ggf. angepassten Lehrplans berücksichtigt werden [5]. Dies betrifft auch Studierende der unteren Semester, die nun vielleicht im Hinblick auf die künftigen Staatsexamina wesentliche praktische Inhalte nicht vollumfänglich erlernen konnten. Hier gilt es, nicht nur in Leipzig zu klären, ob und wie in den kommenden Monaten freiwillige Zusatzangebote oder Refresher-Kurse angeboten werden können, um die evtl. im Rahmen der Pandemie entstandenen Defizite auszugleichen.

Trotz allem hat die SARS-CoV-2-Pandemie dazu geführt, dass lange bestehende Hürden der Digitalisierung überwunden werden konnten und es so zu einem deutlichen Sprung nach vorn gekommen ist. Dieser Umstand wird sich unserer Ansicht nach auch noch in den kommenden Monaten und Jahren weiter positiv auf die Entwicklung des Medizinstudiums auswirken. Sowohl die digitale Weiterentwicklung der Lehrveranstaltungen als auch die Auswirkungen der Pandemie auf die Absolventen sollten in künftigen Untersuchungen näher beleuchtet werden.

## Fazit für die Praxis

- Die SARS-CoV-2-Pandemie stellte die studentische Lehre vor große Herausforderungen. Sie half gleichzeitig aber auch, oft lange bestehende Hürden zu überwinden und der Digitalisierung der Lehre nochmals einen deutlichen Schub nach vorn zu geben.
- Vorlesungen lassen sich auch langfristig gut digital abbilden. Hierbei sollten Lösungen mit der Möglichkeit der direkten Interaktion zwischen Studierenden und Lehrenden bevorzugt werden.
- Veranstaltungen mit direktem Patientenkontakt können gerade in Bezug auf den Erwerb praktischer Kompetenzen digital nicht in Gänze abgebildet werden und sollten frühestmöglich wieder in Präsenz stattfinden. Eine Unterstützung durch digitale Inhalte im Rahmen eines Flipped-Classroom-Konzeptes kann jedoch eine sinnvolle Ergänzung darstellen.
- Die Präferenzen und Sorgen der Studierenden sollten in den kommenden Monaten bei der weiteren Überarbeitung der Curricula von Anfang an mitberücksichtigt werden.

## Supplementary Information




