# Supplementary material for: COVID-19: a chance for digitalization of teaching? Report of experiences and results of a survey on digitalized teaching in the fields of anesthesiology, intensive care, emergency, pain and palliative medicine at the University of Leipzig
Source: Anaesthesist. 2021 Aug 2;71(5):340–9. [Article in German] doi: 10.1007/s00101-021-01016-4 (PMC8326644; doi:10.1007/s00101-021-01016-4)
Supplement: Supplementary file 1 [file 101_2021_1016_MOESM1_ESM.pdf]

Zusatzmaterial zum Beitrag
COVID-19: Eine Chance zur Digitalisierung der Lehre? - Erfahrungsbericht und Ergebnisse einer Umfrage zur digitalen Lehre im Bereich Anästhesiologie, Intensiv-, Notfall-, Schmerz- und Palliativmedizin an der Universität Leipzig -
Hempel G, Weissenbacher A, Stehr SN (2021) in *Der Anaesthesist.*

Beitrag und Zusatzmaterial stehen Ihnen auf www.springermedizin.de zur Verfügung. Bitte geben Sie dort den Beitragstitel in die Suche ein.

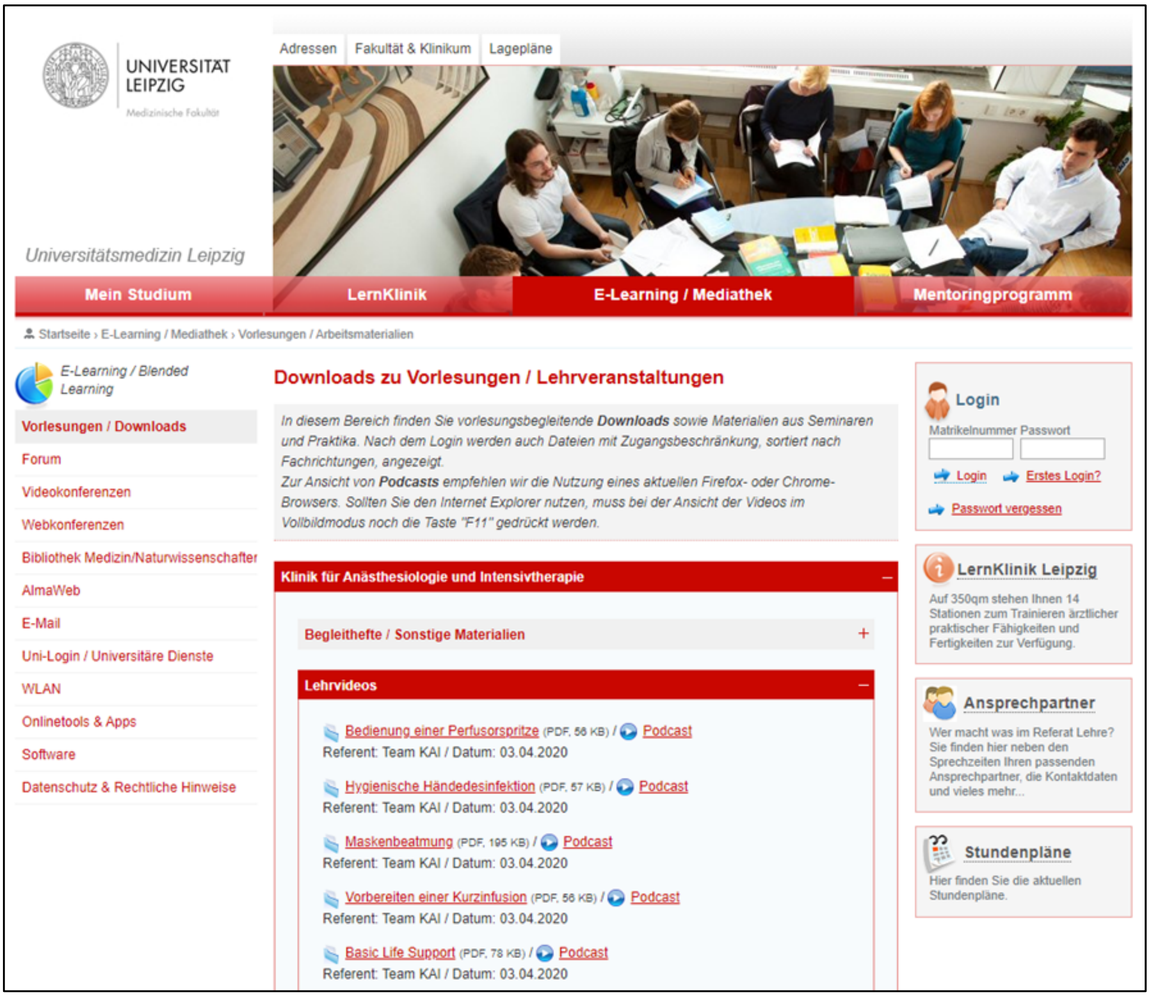

Abbildung 1: Übersicht im Studierendenportal der Medizinischen Fakultät über die Bereitstellung der Lehrvideos der Klinik und Poliklinik für Anästhesiologie und Intensivtherapie (Ausschnitt)

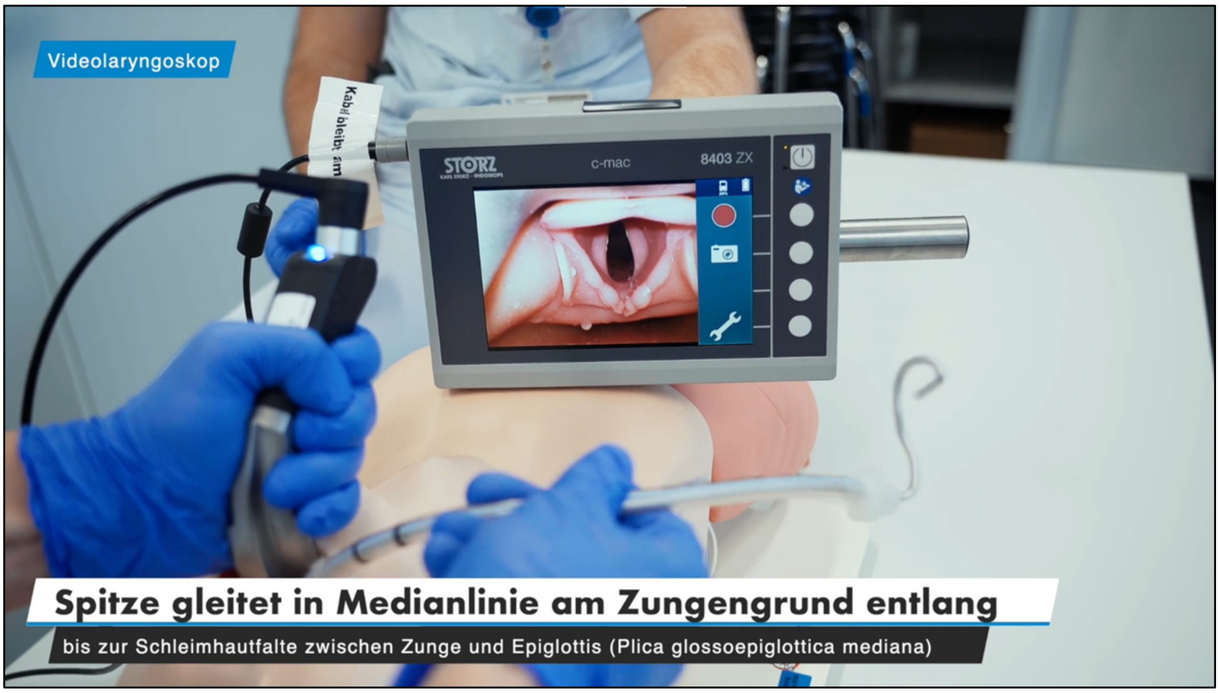

Abbildung 2: beispielhafter Screenshot eines Lehrvideos zur Durchführung der endotrachealen Intubation

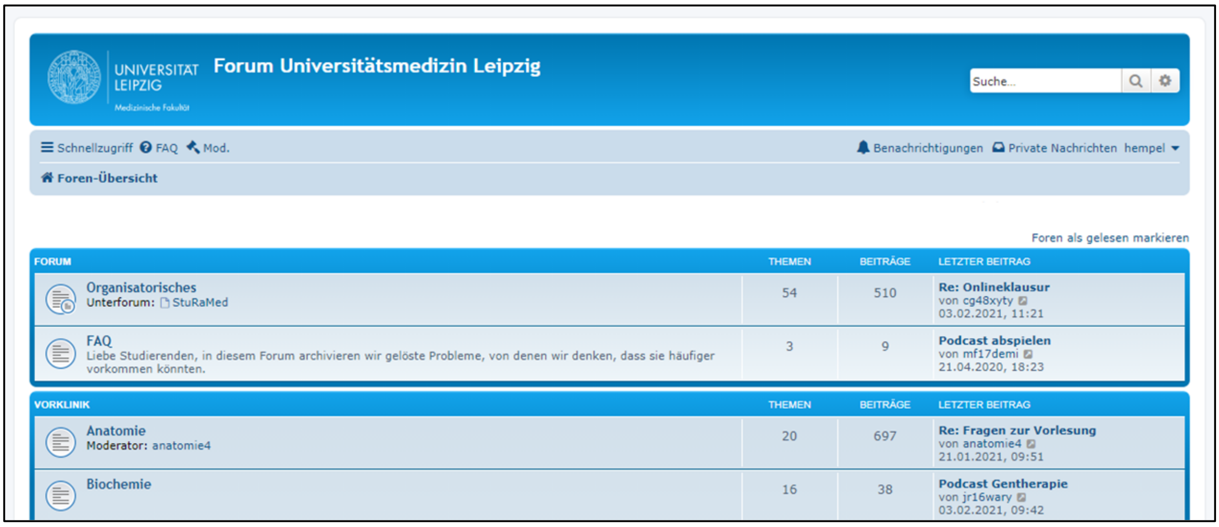

Abbildung 3: Ausschnitt der Forenübersicht des Forums der Medizinischen Fakultät für den Austausch zwischen Studierenden und Lehrenden

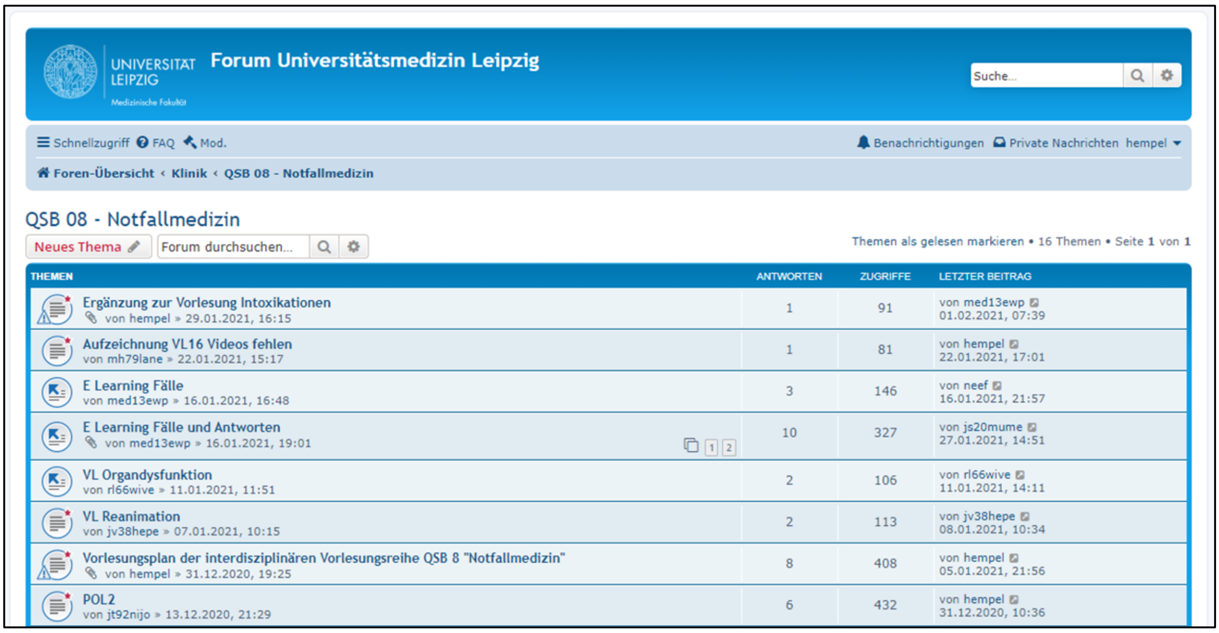

Abbildung 4: Beispielhafter Ausschnitt des Forums "QSB 8 - Notfallmedizin"

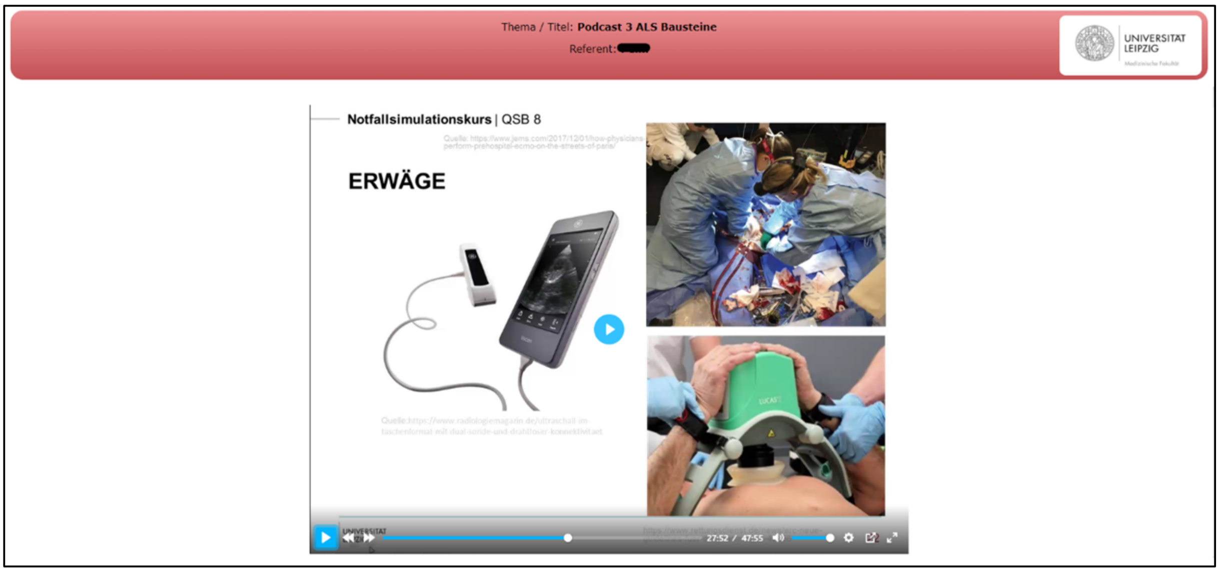

Abbildung 5: Screenshot aus dem Podcast "Advanced Life Support (Teil 2)" zur Vorbereitung auf den digitalen Notfallsimulationskurs
